# Supplementary material for: MetaRibo-Seq measures translation in microbiomes
Source: Nat Commun. 2020 Jun 29;11:3268. doi: 10.1038/s41467-020-17081-z (PMC7324362; doi:10.1038/s41467-020-17081-z)
Supplement: Supplementary file 10 — Supplementary Data 7 [file 41467_2020_17081_MOESM10_ESM.zip › File2/Confidence_High_Taxonomy/106346_out.krona.html]

Javascript must be enabled to view this page.

members
magnitude
magnitudeUnassigned
count
unassigned
taxon
rank

106346\_out

2

superkingdom

SRS893341\_contig\_number\_contig-100\_2464.91017
2
1
2

1239
1
phylum

class
1
186801

1
186802
order

1
186803
family

genus
572511
1

1262756
1

SRS017916\_contig\_number\_contig-100\_1530.114574
species
